# Non-surgical Treatment of Severe Drug-Influenced Gingival Enlargement: A Report of Two Cases

**DOI:** 10.7759/cureus.63214

**Published:** 2024-06-26

**Authors:** Ibrahim Fidan, Edouard Delamotte, Marie-Laure Colombier, Kevimy Agossa

**Affiliations:** 1 Department of Periodontology, University of Lille, School of Dentistry, Centre Hospitalier Universitaire (CHU) Lille, Lille, FRA; 2 Department of Periodontology, Paris Cité University, Montrouge, FRA; 3 Department of Oral Medicine, Louis Mourier Hospital, Assistance Publique – Hôpitaux de Paris (AP-HP), Colombes, FRA

**Keywords:** minimally invasive periodontal therapy, drug-influenced gingival enlargement, cause-related therapy, non-surgical periodontal treatment, amlodipine, gingival overgrowth

## Abstract

Drug-influenced gingival enlargement (DIGE) is a well-known adverse drug reaction associated with multiple medications. Although a benign condition, DIGE can have a significant impact on patients' aesthetic comfort and function. A surgical resection approach is usually proposed to treat severe and generalized DIGE. In this report, we describe the cases of a 47-year-old male and a 58-year-old male, both presenting with severe and generalized DIGE associated with amlodipine, a calcium channel blocker used for hypertension treatment. A non-surgical, cause-related approach, including drug substitution and repeated sessions of mechanical instrumentation, led to the complete resolution of severe DIGE, with no recurrence observed after 18 months in Case 1 and 12 months in Case 2. Throughout the observation period, the bleeding on probing score decreased from 100% at baseline to 10% or less, and the number of periodontal sites with probing pocket depth ≥ 5 mm decreased by more than 90% compared to the initial assessment. Both patients reported a high level of satisfaction with the treatment outcomes. These successful results should encourage clinicians to give greater consideration to non-surgical management of DIGE as a less invasive option before proceeding to surgical treatments.

## Introduction

Drug-influenced gingival enlargement (DIGE) is a well-known adverse drug reaction (ADR), reported in patients using antiepileptic drugs, immunosuppressants, calcium channel-blocking drugs, and high-dose oral contraceptives [1,2]. The prevalence of DIGE ranges widely from less than 5% to more than 80%, depending on the type of drug, patients’ profiles, clinical indices used, and the medical or dental training of the clinician [3]. In a study of the French Pharmacovigilance Database, one-third of DIGE has been linked to calcium channel-blocking drugs, followed by immunosuppressants (15.2%) and anticonvulsants (10.1%) [2].

From a histological point of view, the pathological changes are observed in the connective tissue rather than the epithelial cells of the gingiva. These abnormalities are characterized by an excessive accumulation of extracellular matrix-like collagen with varying amounts of inflammatory infiltrates, predominantly plasma cells. It has been postulated that the inhibitory effect on calcium ion influx across cell membranes in the connective tissue is a key pathophysiological mechanism in DIGE, regardless of the specific drug involved [4,5]. Additionally, bacterial plaque, age, and genetic predisposition may also play a synergistic role in the development of DIGE [6].

Clinically, DIGE can be defined as mild, moderate, or severe, depending on whether the gingival overgrowth is limited to the papilla, or also involves the gingival margin and attached gingiva, respectively [7]. Although a benign condition, DIGE can have a significant impact on patients' aesthetic comfort and function, particularly in severe and generalized forms [8]. Non-surgical periodontal instrumentation along with drug substitution is generally offered as a first-line treatment and can provide some improvement in mild to moderate DIGE. However, resective surgical treatment is usually considered in severe and generalized forms [8]. The clinical efficacy of repeated non-surgical periodontal instrumentation in the treatment of DIGE has been poorly documented [9].

## Case presentation

Case 1

**Figure 1:**
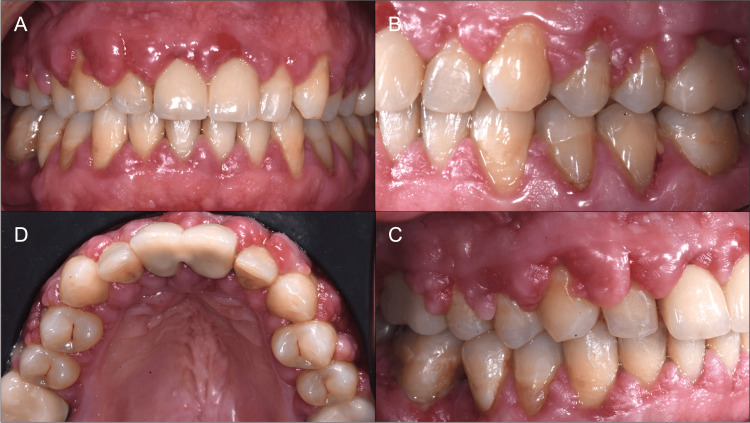
Case 1: initial situation in frontal view (A), lateral views (B and C), and occlusal view (D). Note the presence of clinical inflammation, and the severity of gingival enlargements.

A 47-year-old non-smoking man presented at the clinic of periodontics at the University Hospital of Lille, Lille, France, on January 24th, 2022, with a chief complaint of a generalized gingival overgrowth. His medical history revealed a diagnosis of hypertension nine months ago which was treated with a combination of perindopril/amlodipine 10mg /10mg since April 2021. Gingival symptoms manifested only a few weeks after starting the medication, with complaints of spontaneous gingival bleeding and halitosis. Clinical examination revealed a generalized gingival overgrowth, on both the labial and oral sides of both the upper and the lower arch, involving the interdental papilla, gingival margin, and attached gingiva particularly in the molars (Figure 1).

**Figure 2:**
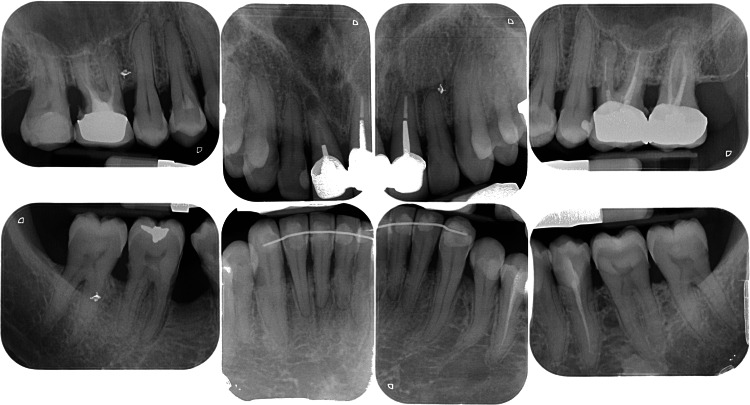
Case 1: initial radiographs. Note the presence of generalized bone loss.

Despite reporting brushing twice a day, the patient exhibited poor oral hygiene. The complete periodontal examination revealed generalized pseudo-pockets (probing depth from 4 to 9 mm) and a bleeding on probing (BOP) score of 100%. Radiographs revealed generalized moderate bone loss on both maxillary and mandibular arches (Figure 2).

A diagnosis of severe and generalized DIGE in Stage 3, Grade C periodontitis patient was established [10], with amlodipine 10mg, a dihydropyridine calcium channel blocker, suspected as the contributing drug.

Case 2

**Figure 3:**
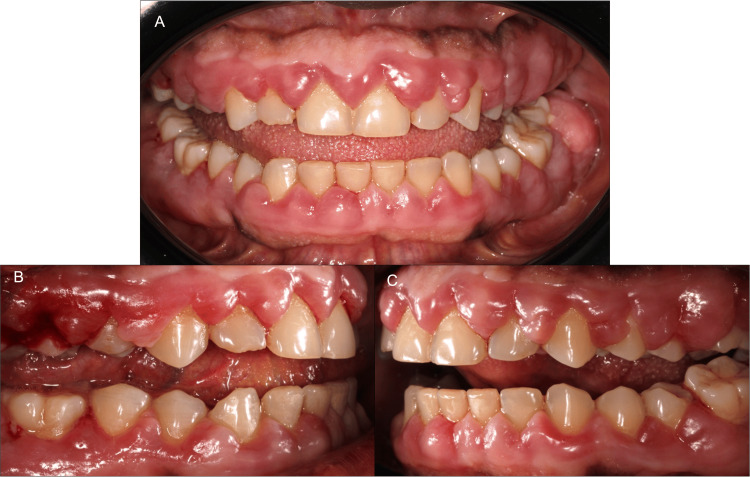
Case 2: initial situation in frontal view (A) and lateral views (B and C). Note the severity of gingival enlargements and the presence of clinical inflammation.

A 58-year-old male patient presented at the Department of Periodontics at Louis Mourier’s University Hospital, Paris, France, on March 1st, 2023, with a chief complaint of gingival overgrowth associated with spontaneous gum bleeding, gingival pain, halitosis, and self-reported tooth mobility. The medical history included hypertension and type 1 diabetes (glycated hemoglobin HbA1c = 7.7%) and the patient had undergone a kidney transplant in 2016. Relevant medications included metformin (antidiabetic drug), amlodipine (calcium channel blocker), tacrolimus, and mycophenolate mofetil (immunosuppressants used to prevent graft rejection). Gingival symptoms manifested a few weeks after starting amlodipine. Clinical examination revealed gingival enlargement similar to Case 1, pseudo-pockets (probing depth from 4 to 12 mm), inadequate plaque control, and generalized inflammation (BOP = 100%) (Figure 3).

**Figure 4:**
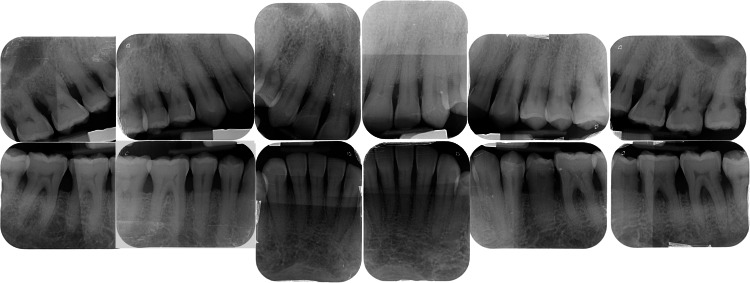
Case 2: initial radiographs. Note the presence of generalized bone loss.

Radiographs also revealed generalized moderate bone loss on both maxillary and mandibular arches (Figure 4).

The diagnosis of severe and generalized DIGE in Stage 3, Grade C periodontitis patient was established [10]. In this case, while amlodipine 5 mg is well known to induce DIGE, mycophenolate mofetil 500 mg is also reported to increase the risk of gingival overgrowth [11].

Clinical management

The treatment was carried out using a stepwise approach following the recent clinical practice guidelines for the treatment of Stage I-III periodontitis [12].

Step 1: Risk Factor Control

Step 1 involved referring the patients to their physician for a switch in medication suspected to be causing gingival enlargement. Additionally, comprehensive oral hygiene instructions were provided, along with a full-mouth supragingival instrumentation using standard ultrasonic tips (Acteon Satelec, Mérignac, France). In Case 1, amlodipine was replaced by Lercanidipine 10 mg and Irbesartan 150 mg, while urapidil 60 mg was prescribed in Case 2.

Step 2: Cause-Related Therapy

Step 2 included multiple sessions of subgingival instrumentation using thin periodontal ultrasonic tips (Satelec Acteon, Mérignac, France). A total of two sessions spaced at two-week intervals were administered. The response to the second step was assessed after a healing period of approximately three months.

Step 3: Complementary Treatments

In Step 3 for the non-responding sites (sites with or without residual bleeding pocket associated with gingival overgrowth), retreatment was carried out through non-surgical repeated subgingival instrumentation only. In Case 1, systemic antibiotics (amoxicillin 1000mg + metronidazole 500mg three times a day for seven days) were administered as adjunctive therapy due to the high number of non-responding sites (deep and bleeding pockets) despite significant improvements in oral hygiene at repeated sessions of mechanical instrumentation in Step 2.

Step 4 

The response to Step 3 was re-assessed after 4-6 weeks and the patients were enrolled in supportive periodontal care every 2 to 3 months for 18 months for Case 1 and 12 months for Case 2.

Results

**Figure 5:**
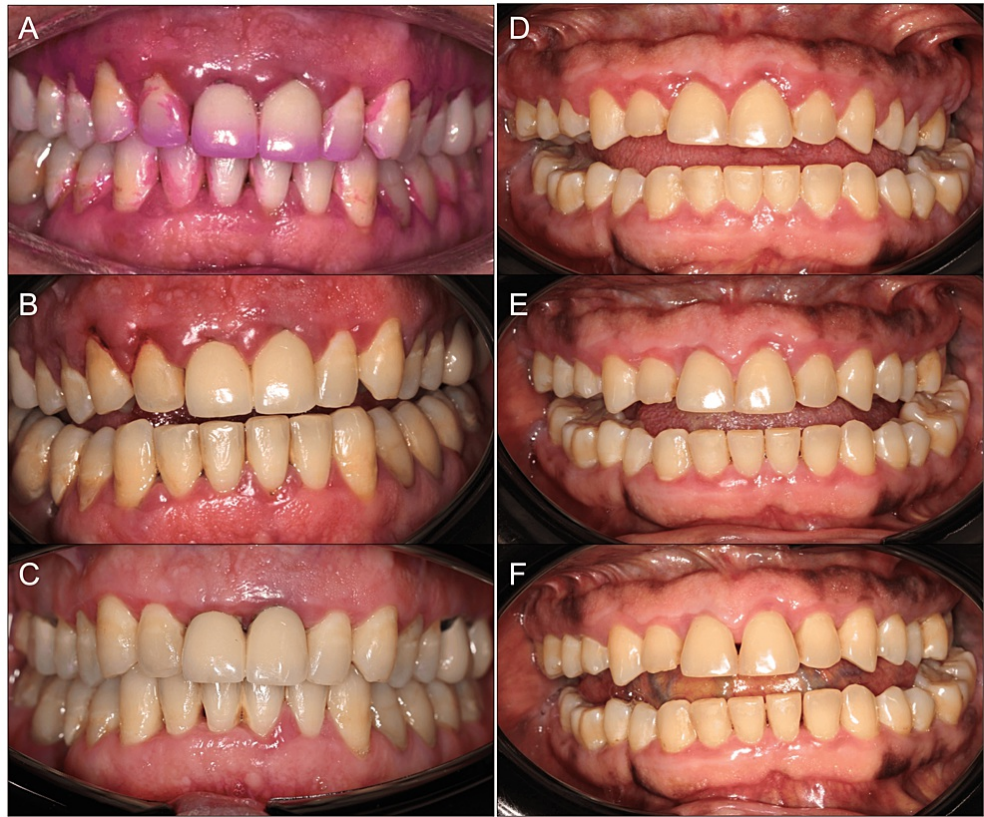
Evolution of the clinical situation during periodontal treatment in Case 1 (A-C) and Case 2 (D-F).

Figure 5 shows the progression of the clinical situation following the initial periodontal treatment. Notably, a significant improvement was evident between three and six months (Figures 5A, 5B, 5D, 5E), with complete resolution of gingival overgrowths achieved between six and eight months (Figures 5C, 5F).

**Figure 6:**
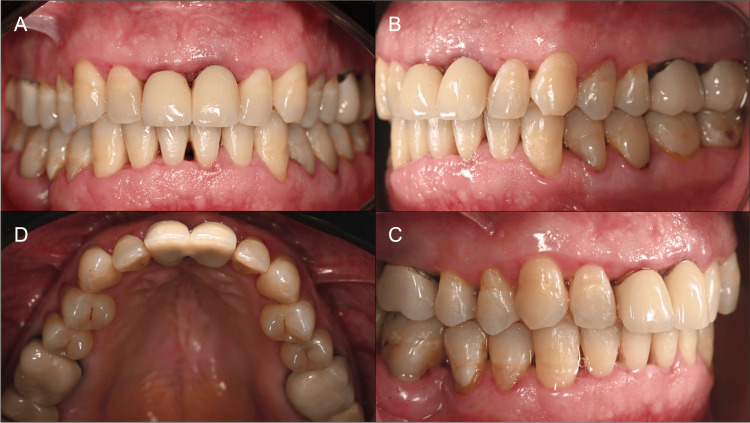
Case 1: situation 18 months after the start of treatment in frontal view (A), lateral views (B and C), and occlusal view (D). Note the complete resolution of gingival enlargements.

**Figure 7:**
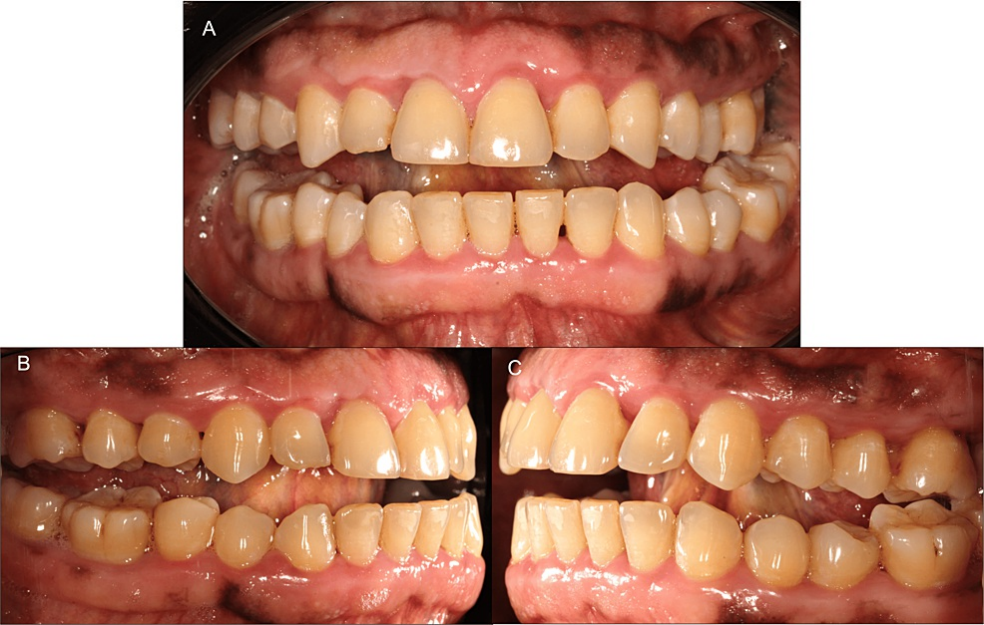
Case 2: situation 12 months after the start of treatment in frontal view (A) and lateral views (B and C). Note the complete resolution of gingival enlargements.

A sustained improvement was demonstrated in subsequent follow-ups, up to 12 to 18 months, with no recurrence of gingival enlargement (Figures 6, 7). 

Throughout the observation period, the plaque score was overall maintained below 30%, and the BOP score decreased from 100% at baseline to 10% after 18 months in Case 1 and from 100% to 7% after 12 months in Case 2. Additionally, the number of periodontal sites with PPD ≥ 5mm decreased dramatically in both cases. Only seven sites in Case 1 and three sites in Case 2 exhibited residual pockets (PPD ≥ 5mm) at the final follow-up appointment, representing a reduction of over 90% compared to the initial assessment. Both patients reported a high level of satisfaction with the treatment outcomes.

## Discussion

Surgical treatment is commonly employed to manage severe DIGE. Indeed, the complete resolution of severe DIGE through non-surgical interventions alone is a rare occurrence, and the value of repeated sessions of supra and subgingival instrumentation to reduce the need for more invasive and costly resective surgical procedures is scarcely documented [9]. It should be noted that in both cases reported herein, the pre-existing periodontal disease, based on bone loss, and multiple local predisposing factors, including inadequate crown forms, open margins, and subgingival calculus must be considered as local risk factors for an exaggerated inflammatory response to medication and dental plaque accumulation [6]. Indeed, in patients presenting with both severe DIGE and periodontitis, the two conditions can exacerbate and accelerate each other, potentially resulting in the loss of the entire dentition [11]. In the cases presented, effective control of local risk factors may have contributed to the favorable response to therapy.

Solid organ transplant patients receiving Cyclosporine A, and who presented incipient DIGE, have demonstrated successful outcomes with early non-surgical periodontal treatment combined with the use of azithromycin or metronidazole [8]. The physiopathology of DIGE is not fully understood, but an increase in extracellular matrix components, particularly interstitial collagen, linked to impaired collagen turnover is reported as a key feature [4,5]. It has been suggested that azithromycin can improve DIGE by inhibiting cell proliferation and collagen synthesis and activating matrix metalloproteinases (MMP-1, MMP-2) in gingival fibroblasts from patients with Cyclosporine A-induced gingival enlargement [13]. Studies have also demonstrated the anti-inflammatory properties of azithromycin or metronidazole, which may explain the positive clinical effect of DIGE [14].

Whenever feasible, withdrawal or substitution of the suspected medication influencing gingival enlargement should be considered the first line and most effective intervention. However, there is almost no evidence regarding the time between treatment modification and the resolution of gingival lesions. A suggested delay of 1 to 8 weeks has been proposed, though not all patients respond to this treatment, particularly those with longstanding DIGE [15]. Based on our experience, it could be speculated that a minimum period of 3 to 6 months after medication switch and non-surgical periodontal treatment is necessary to achieve substantial to complete resolution of DIGE.

One patient presented in this case report (Case 1) received systemic antibiotics as an adjunct to periodontal treatment. Even though the clinical situation improved after a thorough hygiene motivation phase and several sessions of supra and subgingival instrumentation, it was not completely successful as many bleeding deep pockets persisted. In this patient, the combination of amoxicillin and metronidazole was chosen based on current clinical practice guidelines for the treatment of Stage I-III periodontitis [12,16]. While the ideal timing for antibiotic administration during periodontal therapy remains an unanswered question, supragingival plaque control seems to offer substantial clinical and microbial benefits in periodontal patients treated with systemic antibiotics [17]. In the other patient (Case 2), only antibioprophylaxis (amoxicillin 500 mg) was performed before subgingival instrumentation due to the patient's immunosuppression [18]. Interestingly, this patient used mycophenolate mofetil, an immunosuppressant that has been reported to induce DIGE when combined with Cyclosporine A [11]. This drug was not substituted after discussion with the physician because of its benefit-risk balance and good tolerance. However, the favorable gingival outcomes obtained after Amlodipine substitution only could suggest a minor role of this molecule in this patient’s DIGE.

Data on the recurrence of DIGE are limited and may vary depending on the type of treatment. A literature review reports a recurrence rate ranging between 34% and 47.2% within a follow-up duration of 1.5 to 19.9 years [8,19]. It can be assumed that periodic and permanent monitoring, along with periodontal supportive care, plays a crucial role in preventing recurrence. In a recent report, enrollment in a strict maintenance program after non-surgical treatment of DIGE maintained periodontal health for four years of follow-up without recurrence [9].

## Conclusions

This report highlights two successful cases demonstrating the complete resolution of severe DIGE, primarily influenced by amlodipine. The resolution was achieved through a non-surgical approach only, involving drug substitution and repeated sessions of mechanical instrumentation. Not all patients may respond to this treatment modality; however, clinicians may consider this approach as a viable alternative to more invasive and costly surgical treatments, offering potential for successful clinical outcomes and a high level of patient satisfaction.
